# PEACE of mind: Guidelines for enhancing psychological support for medical students

**DOI:** 10.15694/mep.2020.000119.1

**Published:** 2020-06-05

**Authors:** Costas Constantinou, Stelios Georgiades, Alexia Papageorgiou

**Affiliations:** 1 University of Nicosia Medical School

**Keywords:** Medical students, mental illness, PEACE model

## Abstract

This article was migrated. The article was marked as recommended.

The prevalence of mental illness among medical students is high and this continues to be the case in spite of interventions and of providing students with access to mental health services. In this article we present the basic literature on the issue and the reasons why mental illness still prevails among medical students, and we propose the PEACE model for approaching the issue at multiple and interconnected levels, providing examples of relevant actions.

## Introduction

“Medical students must be given better mental health support to prepare them for emotional toll of career in the NHS”.

The above statement is the title of a press release by the British Medical Association (BMA) in 2018. It pinpoints the high rates of mental illness among medical students and it implies that current psychological support is not sufficient for students to develop transferable coping mechanisms for a successful career in medicine. This statement by the BMA highlights an old and ongoing problem which needs to be addressed. In the present paper, we rely on some basic literature to propose the PEACE model of approaching the issue at multiple interconnected levels and filling in this gap. PEACE stands for Professional counseling and support structures, Engagement with social activities and peers, Active mind and psychological wellbeing, and Curriculum Efficiency and academic support. Before presenting the proposed model, it is important to discuss some of the available evidence regarding the magnitude of the problem and why it has not yet been addressed successfully.

## The need to enhance psychological support for medical students

The literature seems to be consistent with regard to mental illness among medical students and many Universities have taken relevant actions to address the increased mental health needs of their students. Based on a systematic review and meta-analysis of depression among medical students, Rotenstein *et al.*’s (2016) explored the findings of 195 studies with 129,123 participants in 47 countries. The study found that the prevalence of depression was 27,2%, while 11,1% of participants had suicidal ideation. Interestingly, only 15,7% of students diagnosed with depression sought professional help. The authors concluded that these were important findings because depression and suicidal thoughts were associated with a risk of suicide in the short-term and depressive episodes and morbidity in the long run. In addition, the same study showed that depressive symptoms were higher among medical students than among individuals of similar age in the general population. More specifically, in the United States, the prevalence was 9,3% among adults aged 18 to 25 and 7,2% among adults aged 26 to 49. The authors explained that high mental illness among medical students largely related to stress and anxiety resulting from a highly demanding and competitive programme.

In support to Rotenstein *et al.*’s study, Karp and Levine (2018, p.1196) explained that about half of the medical students were burnt-out and 10% of them had suicidal thoughts. According to Karp and Levine’s report, medical students had high rates of mental illness because of the demanding nature of the programme, the financial cost and the perceived stigma. Based on such alarming evidence, the authors maintained that medical schools should “revisit their procedures to ensure that they are doing everything possible to meet the mental health care needs of their students”. They carried on presenting the University of Pittsburgh’s model through which psychological support was offered by a mental healthcare team, counseling services were timely and confidentiality was ensured by storing cases away from University hospital’s records.

Along similar lines, a Student BMJ survey in the UK ( Billingsley, 2015) showed that 30% of medical students reported receiving treatment for a mental condition while being medical students. Fifteen percent (15%) of the participants reported having suicidal thoughts. According to Billingsley, having to complete an intense course and stigmatising attitudes were important factors. There were misconceptions about what would happen to the students if they reported mental illness and they tended to avoid seeking help due to the social stigma (see also Constantinou, Georgiou and Perdikogianni, 2017). Billingsley suggested promoting wellbeing and creating an open environment and culture where mental conditions could be discussed without any fear of being stigmatised. The importance of perceived stigma was also highlighted by Janouskova *et al*.’s (2017) cross-sectional study in the Czech Republic with 308 participants. The study found that both students and teachers had stigmatising attitudes towards mental illness and that their attitudes changed after the fourth year; after being introduced to Psychiatry. Billingsley (2015) explained that even doctors and tutors consider mental illness as a form of weakness alluding to personal responsibility for its management. Interestingly, the Student BMJ survey showed that 80% of medical students felt that they were not well supported, they explained that it was not clear to them whom to contact and they were concerned that they were likely to be suspended if they reported mental illness.

As an attempt to address the problem, many Universities have taken actions to reduce stigma and facilitated student access to mental health services ( Slavin, Schindler, and Chibnall, 2014). However, it seems that Universities still need to put more effort as per a press release by the British Medical Association on June 27 2018 entitled “Medical students must be given better mental health support to prepare them for emotional toll of career in the NHS”. The announcement resulted from the last BMA annual meeting which concluded the following:

At the BMA’s annual representative meeting in Brighton, members passed a motion calling for more research into the types of mental health issues experienced by medical students and to improve services available to them. The motion further asks that mental health awareness and promotion of self-care practices are made a core part of the curriculum, and that student health services should provide extended opening hours for those studying medicine, who are often unable to comply with a 9-to-5 timetable.

A few months later the BMA issued a new press release with some basic guidelines for supporting medical students’ and doctors’ psychological wellbeing. To elaborate, the BMA recommended: “build a supportive culture, develop a wellbeing strategy, create healthy workplaces, tackle the stigma around mental ill-health, foster peer support, ensure support services are accessible of high-quality, ensure services have the confidence of those they are intended to help”.

The need to tackle the challenges of managing mental health needs of medical students has also been acknowledged by academics who aimed to propose ways to reduce the problem and place emphasis on the most effective intervention programmes. More specifically, Munn (2017) proposed three approaches, namely reactive (available counseling), proactive (promote wellbeing) and systematic (change culture). Munn explained that changing culture was essential and that this could be achieved by openly discussing mental illness prevalence and management. While programmes of intervention largely reflect Munn“e three approaches, Slavin, Schindler and Chibnall (2014, p.573) explained that curricular changes have not been adequately included in the equation but they could play a positive role. At Saint Louis University School of Medicine, Slavin, Schindler and Chibnall made changes to reduce competition among students (i.e. introduced pass/fail grading scheme), reduced teaching hours and removed detailed material, introduced more electives and developed learning communities (service and advocacy, global health, wellness and medical education) by involving students. They found that these changes “were associated with significantly lower levels of depression symptoms, anxiety symptoms, and stress, and significantly higher levels of community cohesion”. Along similar lines, Wasson *et al*. (2016) explored the effectiveness of 28 interventions tailored for medical students and found that the following programs seemed to be effective: pass/fail grading system, mental health programs, mind-body skills, curriculum structure, wellness programs, and mentoring programs.

Reflecting the findings form the literature, the General Medical Council (GMC) in the UK acknowledged the high rates of mental illness among medical students and published a detailed guide in 2015 entitled “Supporting medical students with mental health conditions”, including recommendations for Medical Schools. The GMC’s report placed emphasis on providing a supportive environment for students. More specifically, “Medicine is an intensive course and some stress is inevitable. But medical schools should try to reduce the pressure put on their students and help them to strike a balance between being committed and being overworked” (p.21). The GMC, therefore, makes a plea for making medical curricula more manageable for students. In addition, the GMC pointed out the following areas that need to be developed: an open culture to mental illness as a way to encourage seeking help and tackling stigma, have support structures in place (Occupational Health and Professional Counseling, personal tutoring, training of staff to know the support structures and identify signs, procedure of supporting students with mental illness and fitness to study), and promotion of well-being.

Although the studies above and the recommendations from the GMC and the BMA were recently published, there is a pending question. Why dealing with the concerning high rates of mental illness among medical students took so long, even though there was some evidence as early as the 1930s ( Slavin, 2016). Slavin analysed a number of false beliefs about medicine which might have contributed to such delay. These are: (a) The belief that medical education has to be demanding to reflect a demanding medical profession. Slavin explained that there was no evidence to support such belief; (b) mental illness was not considered as important and treatable as physical illness; (c) more emphasis has been placed on how to handle symptoms and promote wellbeing rather than on making changes in the curriculum and learning experience.

Despite the abundance of research findings and the attempts of many Universities to address these issues, prevalence of mental illness among medical students is still high ( Rotenstein *et al.*’s, 2016). There is not much information in the literature to confidently answer why the problem has not been reduced. According to Slavin, Schindler and Chibnall (2014, p.573), this can be attributed to the fact that the provision of access to mental health services and the reduction of stigma might not be enough for reducing the prevalence of mental illness among medical students and that curriculum changes are imperative. In addition to Slavin, Schindler and Chibnall’s findings, another factor which has not been well integrated in previous recommendations and can contribute to the students’ psychological wellbeing is that of social engagement. Lim *et al.*’s (2016) systematic review revealed a link between mental illness and social isolation and that loneliness was associated with poor quality of life. Reflecting these findings, Thomas *et al*. (2017) reported on student retention and success. Drawing information from thirteen Universities in the UK, they found that social engagement and belonging were among the key factors contributing towards retention. Lawther and Foster’s (2013) study at Nottingham Trent University found that more than 30% of students considered not continuing with their studies and explaining that poor sense of belonging was the driving force. The three more important reasons for not belonging were “don“t get along socially”, “struggled with work” and “no longer interested in the course”.

On the basis of the information reviewed thus far it seems that a multilayered approach is needed with simultaneous actions at different levels in order to successfully tackle the issue of mental illness among medical students. The enhancement of psychological support for medical students can be achieved at four different but interconnected levels, namely addressing the curriculum demands, psychological well-being, social engagement and support structures. We are turning these four levels into a circular model for better enhancing psychological support for medical students as presented below.

## PEACE of mind: Guidelines for enhancing psychological support for medical students

**Figure 1.**
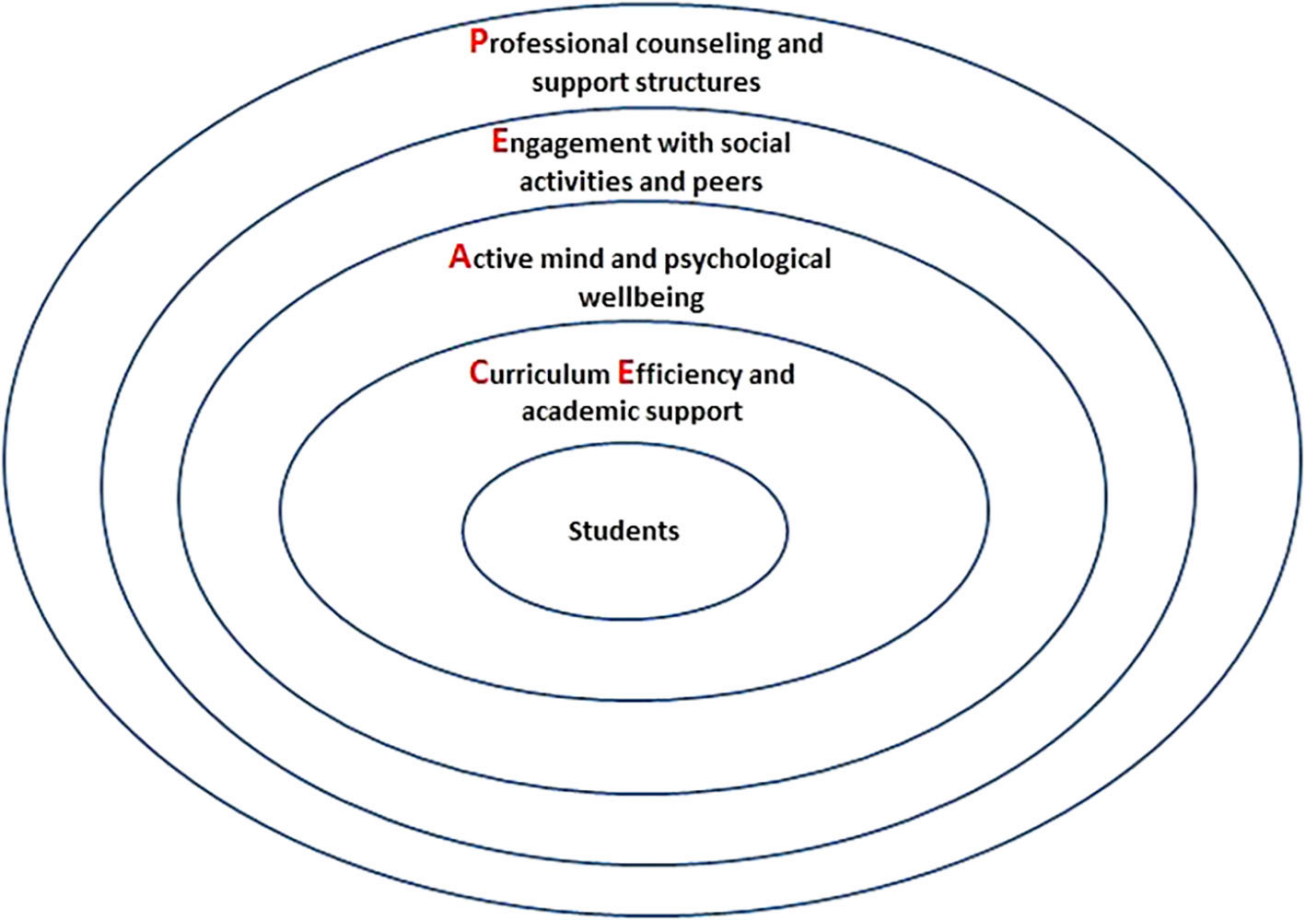
PEACE model

Relying on the basic literature and GMC and BMA recommendations, we propose the student-centred PEACE circular model ( Figure 1) for approaching the issue of enhancing psychological support for medical students. PEACE is the abbreviation for **P**rofessional counseling and support structures, **E**ngagement with social activities and peers, **A**ctive mind and psychological wellbeing, and **C**urriculum **E**fficiency and academic support. PEACE could potentially help students stop experiencing exaggerated worry about succeeding in a highly demanding course; hence providing a “PEACE of mind”. It also means that a successful implementation of the model could potentially create a more enjoyable and a safer environment for student development.

Here we present the rationale of the circles’ positions. As per Figure 1, students are in the centre of the model as they are the target of support and development. The circle next to the students is the curriculum efficiency because a course is among the main reasons why students chose to attend a University and completion of a degree is their goal for the years to come. So the course is in the closest proximity to being a student. Therefore, a course that is not overwhelming, that is enjoyable and provides students enough time for other activities is essential. In the outer circle is students’ ability to develop and maintain an active mind and wellbeing. This has to do with individual cognitive actions and behaviours which can help them cope with the demands of the course but also with increasing their social participation. The next circle refers to social engagement which is outside personal space whereby students can reach out to participation in a social sphere within the context of activities or with their peers. Social engagement could help both maintain an active mind but also have breaks from studying. The last circle is professional counseling and other support structures because they can have an overarching role for helping students’ success in all the previous circles but also provide the necessary support in case students experience psychological symptoms or develop a mental condition. Let us now discuss each circle in some more detail and present basic guidelines or examples of actions.

### Curriculum Efficiency and academic support

Curriculum efficiency and academic support are key factors for student retention and success. Based on Slavin, Schindler and Chibnall’s (2014) study findings, a medical curriculum could achieve efficiency by adopting a student-centred learning approach whereby students learn how to apply their knowledge and skills, employ active learning, learn in groups, and develop as life-long independent learners with transferable skills. Student-centred learning was inisitutionalised in EU with the Bologna process in 2009 and research evidence has shown that it enhances student learning and development ( Kaput, 2018). The European Student Union has highlighted the importance of implementing student-centred education explaining the benefits for both students and the Universities ( Attard *et al*., 2010). Developing life-long transferable learning skills instead of merely acquiring textbook-like knowledge could address the challenge of having an overwhelming material to handle and exerting pressure on students to succeed. Examples of relevant actions:

• Simplify textbook-like material, remove detail or unnecessary information and reduce contact hours.
• Deliver teaching that facilitates understanding by adding more case-based scenarios for applying taught material and for self-directed learning.
• Incorporate active learning (work with cases, in groups, projects etc).
• Provide timely academic support.
• Provide student feedback which can help them develop their life-long learning skills.
• Involve students as partners in the development of curriculum.
• Remove competition from assessment and marking schemes.
• Discuss mental illness and fitness to study within the curriculum.

### Active mind and psychological wellbeing

Niemi and Vainiomäki (2006) study showed that medical students reporting stress increases over years of study for both males and females. The authors suggested medical students being trained in stress management in order to facilitate their transition to the University but also to cope with challenging situations within the context of a demanding academic degree. Machato *et al*. (2019)’s study reached similar conclusions by showing that promotion of psychological wellbeing to medical students improved life satisfaction and positive emotions and decreased symptoms of mental illness. Echoing such research findings, both the GMC and the BMA formulated clear recommendations clarifying that promotion of wellness is essential for equipping students with tools to cope with psychological challenges. Psychological wellbeing also reflects WHO’s definition of health and published recommendations in how to develop one’s psychological wellbeing. On this note, students could be trained in how to develop and maintain an active mind and practice psychological wellbeing mechanisms. Examples of relevant actions:

• Organise interactive sessionsto inform students about the prevalence of mental conditions among medical students and that mental illness is quite common and treatable.
• Train students in recognising the symptoms and seeking help, and promote wellness and self-care.
• Train students in stress management, time management skills, study skills and learning how to accept and develop from personal failures.
• Establish relevant clubs and societies (e.g. wellness, mental health group etc)
• Introduce or enhance a Personal Tutor (PT) scheme. Personal tutors can support their tutees, help them cope with minor symptoms, refer them to a professional, help tackle perceived stigma.

### Engagement with social activities and peers

Social engagement is important for contributing to both psychological wellbeing and general quality of life. Lim *et al.* (2016) showed how social isolation is linked with mental illness, while Thomas *et al.* (2017) and Lawther and Foster (2013) stressed the importance of social engagement and the sense of belonging for student retention and success. Social participation could help students feel more integrated into the student society, reflecting wider research findings on inequalities and health which show that social integration is associated with better physical and mental health ( Brydsten, Rostila and Dunlavy, 2019). Social engagement, thereafter, in the PEACE model is before professional counseling as it can be a protective factor and could take many forms. Examples of relevant actions:

• Social activities which reflect student needs. Social events could be organized by both Student Unions and Student Services.
• Increase student participation in clubs and societies.
• Design programmes for meaningful peer relations and student/ staff interactions.
• Reward social engagement by introducing extra-curricular activities awards scheme. This is important for encouraging students to socially engage but also to enhance their curriculum vitae.

### Professional counseling and support structures

When crisis strikes students need help. Therefore, good quality, timely and confidential professional help is fundamental, as per the GMC’s and the BMA’s guidelines. In the PEACE model, professional counselling is in the outer circle of the model not because is the least important but because it is crucial for supporting the other circles as well as for helping students overcoming any mental challenges and difficulties. More specifically, counseling can provide useful advice for developing active mind and psychological wellbeing techniques as well as study skills and ways to be socially engaged. On the other hand, professional counselling should be confidential and timely for the students who experience psychological difficulties or specific psychological symptoms. Supporting structures should also encompass the mechanisms for ensuring fitness to study and for a successful reintegration in the course in case a student takes time off for psychological treatment. Examples of relevant actions:

• Provide confidential and timely professional counseling.
• Establish efficient structures and procedures for Occupational Health, fitness to study and reasonable adjustments.
• Train both faculty and admin staffin recognising mental illness symptoms and communicating with students effectively.
• Support students to return to the course from psychological or psychiatric treatment.

## Critical reflection on PEACE

As a framework or pathway for action, the PEACE model has some important advantages. First, it reflects the basic literature and recommendations and it has been informed by research evidence which shows that curriculum changes, psychological wellbeing, social engagement and support structures are protective pillars for medical students’ psychological welfare. Second, the model is not a random presentation of suggestions. It focuses on four levels which are reasonably positioned in relation to their distance from the students as individuals. More specifically, it is not accidental that we have not suggested that professional counseling should be the first level that students should be exposed to. Counseling and other support structures are essential to handle crises and psychological problems when they occur and provide support to other structures. However, before students seek or referred to professional help, the other three levels could work as preventive contexts. Third, the importance of social engagement is highlighted in the model, while it seems to be absent from or underdeveloped in other recommendations and guidelines which focus largely on counseling and promotion of psychological wellbeing. Fourth, the model is easy to implement as the four levels could be independently managed by different departments.

There are also challenges to consider. The model needs to be tested as a whole or as individual levels of intervention in order to better understand which areas work better for reducing the rates of mental illness among medical students. Another challenge is that of student diversity. University students make a group with diverse learning styles, experiences, ambitions and goals, and cultural background. Diversity could be addressed within each level through training staff in how to deliver teaching to a diverse audience (curriculum efficiency), introduce a variety of mechanisms for psychological wellbeing (active mind and psychological wellbeing), increase the range of clubs and societies and organise events that reflect such student diversity (engagement with social activities and peers), training counselors and other professionals in how they can provide cultural competent care and support (professional counseling and support structures).

## Conclusion

In this article we have presented a long-standing challenge for medical students and medical schools which relates to the high prevalence of mental illness. Although the problem was identified as early as in the 1930s it was only recently that systematic actions were taken and guidelines were formulated. Despite intervention programmes and facilitating student access to mental health services the problem still remains. In order to address the issue, we reviewed some basic literature and formulated the PEACE model which adopts a multilayered approach to students’ mental health, suggesting guidelines and actions at four levels, namely curriculum efficiency, active mind and psychological well-being, social engagement and psychological counseling and support structures. More systematic intervention programmes could be designed at all these levels and tested either individually or all together in order to gauge their impact on students’ psychological well-being.

## Take Home Messages

• Mental illness among medical students is high.
• Universities should take actions to reduce the rates of mental illness among medical students and support students appropriately.
• The PEACE of mind model approaches the issue at multiple and interconnected levels, providing examples of relevant actions.

## Notes On Contributors

**Costas S Constantinou** is an Associate Professor of Medical Sociology and Associate Dean for Students at the University of Nicosia Medical School. His research interests are illness experience, illness narratives, organ donation and transplantation, ageing, student-centred education, cultural competence, and qualitative research methodology.

**Stelios Georgiades** is an Assistant Professor of Clinical Psychology at the University of Nicosia Medical School. His research interest is on the role of schizotypy in the deveoplent of schizophrenia and the efficacy of Cognitive Behavioural Therapy in the treatment of schizophrenia and post-traumatic stress disorder. He is also interested in the investigation of the role of psychology in medical practice and in psychological education.

**Alexia Papageorgiou** is a Professor of Clinical Communication and Chair of the Centre of Medical Education at the University of Nicosia Medical School. Her research covers the areas of clinical communication, medical education, health psychology and advance statements of people who can no longer make decisions in medicine and psychiatry.
