# Supplementary material for: PEACE of mind: Guidelines for enhancing psychological support for medical students
Source: MedEdPublish (2016). 2020 Jun 5;9:119. [Version 1] doi: 10.15694/mep.2020.000119.1 (PMC10718069; doi:10.15694/mep.2020.000119.1)
Supplement: Peer review history

# Reviewer report: Reviewer response for version 1

**DOI:** 10.21956/mep.20051.r30736

**Published:** 2020-10-20

**Authors:** Ken Masters

**Affiliations:** 1 Sultan Qaboos University

This review has been migrated. The reviewer awarded 3 stars out of 5 An interesting paper proposing guidelines for enhancing psychological support for medical students. Through the literature, the authors establish firmly that medical students’ mental health issues are cause for concern. Although these issues appear to have been previously acknowledged, and Universities have taken steps to address them, these steps have not been entirely successful, and the delays in acknowledging the problems have compounded them.In response, the authors propose a model. While the model is interesting, I’m not sure that it adds a great deal to what we already know; it does have the advantage, however, of combining much of what we know into a single approach. The activity examples proposed are interesting, and certainly make sense from a purely logical point of view, but the authors have not supported those with studies. What I mean is this: In each area, the authors have established that there is a need, and that is done (mostly) based on literature. Then they propose activities to meet those needs. But, there is nothing to support the idea that those activities will, however, support that need. The authors do acknowledge that the model has to be tested, so that would be a good starting point – to see the extent to which the proposed activities (and other activities) meet the needs as described.So, I would like to see a version 2 of this paper in which the activities are a little more strongly supported by the literature.Beyond the scope of this paper, I would also like to further research papers in which these are investigated in more detail.

Reviewer Expertise:

NA

No decision status is available

# Reviewer report: Reviewer response for version 1

**DOI:** 10.21956/mep.20051.r30734

**Published:** 2020-06-11

**Authors:** Wafaa Mowlabaccus

**Affiliations:** 1 University of Mauritius

This review has been migrated. The reviewer awarded 4 stars out of 5 Being a medical student from a developing country, I find this article very enriching and educational. While there are scant support provided for mental wellbeing of medical students in my country, I suggest the implementation a framework based on the PEACE model which can be globalized.

Reviewer Expertise:

NA

No decision status is available

# Reviewer report: Reviewer response for version 1

**DOI:** 10.21956/mep.20051.r30732

**Published:** 2020-06-11

**Authors:** Hebat Allah A. Amin

**Affiliations:** 1 Department of Pathology, Helwan University, Faculty of Medicine

This review has been migrated. The reviewer awarded 4 stars out of 5 An important topic that needs to work promptly on it. The presented PEACE model ans the suggested multilayered approach are greatly appreciated as a possible guide for the Faculties' students support centers. I hope you can publish case studies on this urgent matter.Your work is much appreciated.

Reviewer Expertise:

NA

No decision status is available

# Reviewer report: Reviewer response for version 1

**DOI:** 10.21956/mep.20051.r30735

**Published:** 2020-06-11

**Authors:** Nagwa Hegazy

**Affiliations:** 1 Menoufia university

This review has been migrated. The reviewer awarded 4 stars out of 5 Interesting article specifically nowadays with all of theses COVID-19 stressors.The problem is usually in the stigma & definitely prevention is a better step.I think PEACE model is a good trial for that. Further research on implementation and challenges is highly advised.

Reviewer Expertise:

NA

No decision status is available

# Reviewer report: Reviewer response for version 1

**DOI:** 10.21956/mep.20051.r30731

**Published:** 2020-06-11

**Authors:** Shamaila ijaz

**Affiliations:** 1 Lisca

This review has been migrated. The reviewer awarded 4 stars out of 5 A very thoughtful approach and need of the day especially in this time of COVID. A sample study could have been a better approach.

Reviewer Expertise:

NA

No decision status is available

# Reviewer report: Reviewer response for version 1

**DOI:** 10.21956/mep.20051.r30733

**Published:** 2020-06-07

**Authors:** P Ravi Shankar

**Affiliations:** 1 American International Medical University

This review has been migrated. The reviewer awarded 4 stars out of 5 This is an interesting article on a very important issue of psychological well-being of medical students. This is an issue which does not often receive the attention it requires. In South Asia, especially the stigma against mental illness is very strong and students are extremely reluctant to seek help. I have experienced the deleterious effects of mental illness among peers when I was a student and among students when I was a faculty member. The PEACE model makes sense and provides an organized framework to deal with the problem. The issue of diversity of the student population is important. As I had mentioned some groups are more reluctant to seek help and support compared to others. Hence counselling and support may have to be provided in a culturally appropriate manner. Personal counselling and having a mentor is effective to reduce distress and provide support. Most of the authors’ citations and references are from the United Kingdom. Is there any particular reason for this as the medical school and the authors are from Cyprus? The article will be of broad interest.

Reviewer Expertise:

NA

No decision status is available
